# Prevalence of lip lesions in an Indian population

**DOI:** 10.4317/jced.51597

**Published:** 2014-10-01

**Authors:** Santosh Patil, Sneha Maheshwari

**Affiliations:** 1 Dept of Oral Medicine and Radiology, Chattisgarh Dental College and Research Institute, Rajnandgaon (Chattisgarh). India; 2 Dental Practitioner, Jodhpur (Raj). India

## Abstract

Objectives: Lip lesions are a frequent cause of consultation in dentistry, as they comprise a significant proportion of the oral lesions. The aim of the present study was to identify the different lip lesions and determine their prevalence in an Indian population. Material and Methods: 5231 patients visiting a Department of Oral Medicine and Radiology were examined for the presence of different lip lesions during the period from October, 2011 to May, 2013. The statistical analysis was done using Chi-square test and p<0.05 was considered to be statistically significant. Results: The age of the patients ranged from 8-70 years with a mean age of 32.6 years. The prevalence of lip lesions was 18.8%. The most commonly diagnosed lesions were those due to infections, which affected 32.6% of the population, followed by mucocele (29.8%) and premalignant lesions and conditions which were observed in 20.6% of the population. Males were more commonly affected than females. Conclusions: The relatively high prevalence of the lip lesions suggests dental practitioners and health care workers to educate the patients and create more awareness regarding them. The dentists should have adequate knowledge about the etiology, clinical features, diagnosis and management of the lip lesions.

** Key words:** Lip, lesions, India, prevalence.

## Introduction

Lips are highly exposed to various factors such as ultraviolet radiations [UV], food, tobacco among others that may result in substantial morbidity and rarely, mortality of the patients due to their anatomical location. These obvious abnormalities are a cause of concern for the patients primarily due to esthetic considerations. Diagnosing and treating lip lesions are important not only to prevent morbidity and mortality, but also to avoid any embarrassment to the patient in social gatherings and to maintain the patient’s self-esteem (1).

A lip lesion may result due to biting or injury or may appear as a symptom of an infection or other underlying medical condition which might include digestive disorders, inflammatory conditions, or cancer. Clinicians regularly encounter lip lesions in routine practice. A thorough knowledge of the lesion, proper history recording and clinical oral examination aids in identifying and diagnosing the lesion. Various epidemiological studies have been done around the globe reporting the prevalence of oral mucosal lesions in different populations (2-6). However, fewer studies have been done to estimate the prevalence of lip lesions. One such large scale study has been reported in the oral screening clinics at Minnesota, U.S.A. (7). No such study has been done in the Indian subcontinent. The present study was designed to study the prevalence of the lip lesions in an Indian population.

## Material and Methods

5231 patients attending the outpatient clinic of the Department of Oral Medicine and Radiology, Jodhpur Dental College General Hospital were examined for the presence of various lip lesions during the period from October, 2011 to May, 2013. All the patients of the age range from 8-70 years attending for various complaints were examined. Ethical clearance was obtained from the Institutional Ethical Committee. A written informed consent was also obtained from the patients. A detailed family and medical history along with the history of any associated habits such as tobacco, smoking, and alcohol consumption was recorded. The clinical examination was done under artificial illumination on a dental chair. The lips were examined for any change in surface texture, colour, size and specific lesions. Lymph nodes were also examined. The lesions were grouped under the following categories:

1. Developmental: Cleft lip, Fordyce spots, double lip, congenital lip and commissural pits and fistulas, macro-cheilia, microcheilia, eclabium.

2. Infection: Viral: herpes simplex/Recurrent herpes labialis

Bacterial: Staphylococcus aureus [Impetigo]

Fungal: Candida albicans [Angular cheilits]

3. Granulomatous disorders: Sarcoidosis, Crohns disease, cheilitis granulomatosa, tuberculosis.

4. Autoimmune disorders: Systemic lupus erythematosus [SLE].

5. Inflammatory conditions: Cheilitis glandularis, exfoliative cheilitis, contact cheilitis, chronic atrophic-abrasive cheilitis, eczema around lips.

6. Allergic: Angioneurotic edema, Erythema multiforme.

7. Salivary gland disorder: Mucocele

8. Pigmented lesions: Hemangioma, lentigo, labial melanotic macule

9. Premalignant lesions and conditions: Leukoplakia, oral submucous fibrosis [OSMF], actinic cheilitis, lichen planus

10. Benign tumours: Fibroma, papilloma, other.

11. Malignancies: Squamous cell carcinoma, basal cell carcinoma

Most patients were aware of the lesions and presented with various associated symptoms such as pain, ulcerations, appearance of the lip, presence of any overgrowths, difficulty in speech and esthetic concerns. None of the patients were taking any medications for the lesions. Histopathological examination was required in few cases to confirm the clinical diagnosis. The data was collected and analyzed using SPSS 12.0. The statistical analysis was done using Chi-square test and *p*<0.05 was considered to be statistically significant.

## Results

The study comprised of 5231 subjects, of which 2678 were males and 2453 females. The age of the patients ranged from 8-70 years with a mean age of 32.6 ± 6.2 years. The mean age of males was 33.4 ± 6.8 years and of females was 31.9 ± 5.7 years. Of the total patients examined, 984 presented with various lip lesions with a prevalence of 18.8%. Males [629 patients; 63.9%] were more commonly affected than females [355 patients; 36.1%] but this was not statistically significant [*p*>0.05]. The distribution of the various lip lesions is shown in  Table 1. The most common lesions diagnosed were due to infections which affected about 32.6% [321 patients]. Mucocele was observed in 292 patients [29.8%], premalignant lesions and conditions in 203 patients [20.6%]. Lip lesions due to immunological disorders and pigmented lesions such as hemangioma, lentigo and labial melanotic macule were the least common lesions, seen in only 0.2% and 0.4% of the studied population respectively.

**Table 1:**
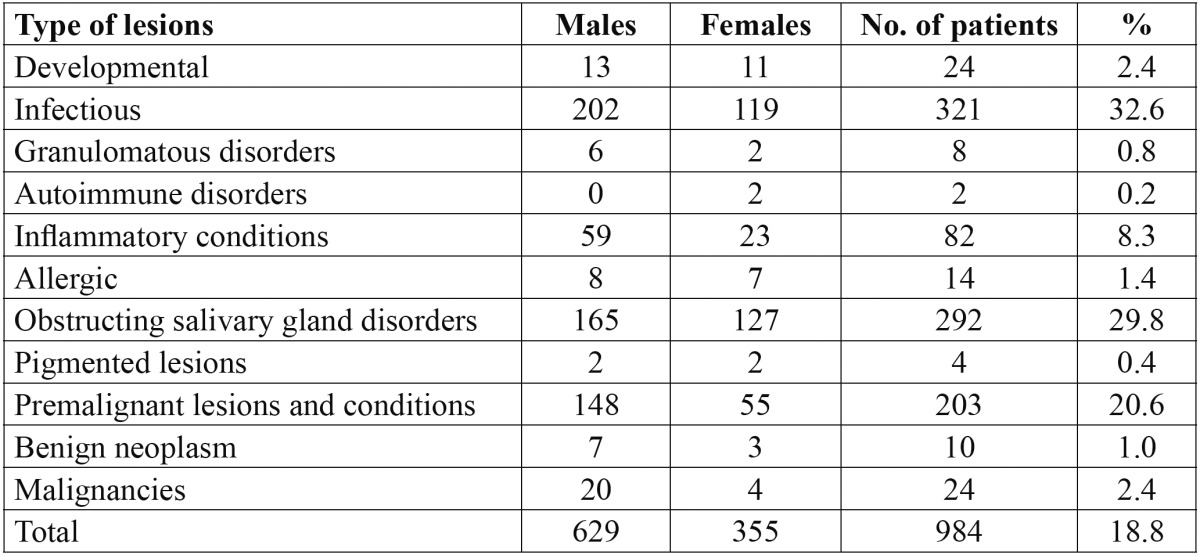
Distribution and prevalence of various lesions of the lip.

## Discussion

Lips are anatomically situated in a very prominent position and play important role in manipulation of food and phonetics, and may be affected by a wide variety of diseases. Most of these disorders may present with characteristics and may be easily identified, but many lip lesions can present a diagnostic and therapeutic dilemma for the dental practitioners. Early identification and diagnosis can be done by a thorough history of the lesion, preceding symptoms, and related habits, if any. Cleft lip may be seen due to developmental defects (8). Cheilitis glandularis is a chronic inflammatory disorder which affects the labial salivary glands and their ducts. Cheilitis granulomatosa may be seen as one manifestation of orofacial granulomatosis. The granulomatous conditions of Melkersson-Rosenthal syndrome, sarcoidosis, and Crohn’s disease may be associated with cheilitis granulomatosa or it may stand alone as Miescher’s cheilitis. Intestinal dysfunction can also cause improper nutrient absorption in celiac disease, of which lip lesions are a primary indicator. Angular cheilitis, is a reactive process which is usually seen bilaterally at the corner of the mouth and may have variable etiological possibilities like infections, mechanical trauma, nutritional deficiencies, and diverse dermatoses. Contact cheilitis may occur because of a primary irritant or a delayed hypersensitivity reaction. Exfoliative cheilitis is a reactive condition secondary to factitious activity of the patient (9-11). Apart from these conditions various malignant and pre-malignant conditions such as squamous cell carcinoma, basal cell carcinoma, leukoplakia and OSMF may affect the lips (12).

There has been only one large scale study done to estimate the prevalence of lip lesions, performed in the oral screening clinics at Minnesota, which reported a prevalence of 29.6 per 1000 patients (7). However, certain other prevalence studies which included individual lip lesions along with other lesions of the oral cavity have been reported (3-5). The present study was aimed at estimating the prevalence of various lip lesions in an Indian population. The prevalence of lip lesions in the present study was estimated to be 18.8%, which is much higher than the findings of Bouquot *et al.* (7). Reported differences may be due to variations in the ethnicity, geographical differences, design of the study [in general population vs in a clinical space], diagnostic criteria used for the study, and gender variations in the study samples.

The most common lesions in the present study were of infectious nature [32.6%], resulting due to various viral [herpes simplex/Recurrent herpes labialis], bacterial [Staphylococcus aureus [Impetigo]] and fungal [Candida albicans [Angular cheilits]] infections. Most common of these lesions were due to viral infections, which comprised about 64% of the infectious lesions. Bouquot *et al.* (7) reported a prevalence of 2.5 of herpes labialis and 1.9 prevalence [per 1,000 persons] of angular chelitis.

Angular cheilitis can occur spontaneously but is seen commonly in denture wearers and those with orthodontic appliances, those who wear masks as part of their occupation, and in some small children, who slobber and use pacifiers. It is also common among HIV-infected patients and patients with Down syndrome (13,14). The children who frequently lick their lips and suck their thumbs have also a high prevalence of this lesion. Excessive mouth washing and aggressive dental floss use may contribute to its development (15).

The second most common lesion was mucocele which was present in 29.8% of the studied population. This was much higher than the findings of Bouquot *et al.* (7), who reported a prevalence of 1.4 [per 1,000 persons]. Trauma to minor salivary glands has been quoted as the prime reason that lead to the development of an extravasation mucocele. The most common site of occurrence is the lower lip, where the history of trauma has been commonly reported (16).

Premalignant lesions and conditions were the third most common lesion with a prevalence of 20.6%. It was however the most common lesion in the study conducted by Bouquot *et al.* (7) with a prevalence of 9.3/1,000 persons. Leukoplakia can occur at any site of the oral cavity, including the vermillion border of the lip, which is the most frequently involved site of the lip. It has a relatively high rate of malignant transformation due to its strong correlation with tobacco and alcohol use (1,7). Oral lichen planus is a relatively common disorder which occurs commonly on the buccal mucosa, but may also involve the gingiva, tongue, floor of the mouth and retromalar pads. Isolated lichen planus of the lip is unusual and is usually seen in association with oral lesions. The prevalence of lichen planus as reported by Bouquot *et al.* (7) was 0.13/1,000 persons. The lesion presents a diagnostic dilemma for the clinicians and requires an appropriate differential diagnosis (17). Actinic cheilitis is a premalignant, irreversible disease that frequently affects the vermilion border of the lower lip. A total of 10 cases [4.9%] of actinic cheilitis were reported in the present study. This was low when compared to the findings of Martins-Filho PR *et al.* [16.7%] (18), de Souza Lucena EE *et al.* [15.5%] (19) and Miranda *et al.* [9.16%] (20).

The inflammatory lesions were observed in 8.1% of the population, which comprised of cheilitis glandularis, exfoliative cheilitis, contact cheilitis, chronic atrophic-abrasive cheilitis and eczema around lips. Cheilitis glandularis is an uncommon disease usually involving the lower lip of adults and is characterized by enlargement and eversion of the lip in association with excretory duct dilatation (21). In some cases, it may degenerate into squamous cell carcinoma (22). Cheilitis granulomatosa is a rare inflammatory disorder characterized by recurrent or persistent swelling of one or both lips. It is a manifestation of orofacial granulomatosis, which describes facial and oral swelling in cases of non-caseating granulomatous inflammation and in the absence of systemic disease such as Crohn’s disease and sarcoidosis. The lesion can occur by itself or as part of the Melkers-son-Rosenthal syndrome. Clinical history is important in diagnosis of the lesion, as similar orofacial swelling may be an early manifestation of Crohn’s disease or sarcoidosis (23). Lesions associated with granulomatous disorders were reported in 7 patients with a prevalence of 0.8%.

Developmental lesions were observed in 2.4% of the population. Clefts of the lip and/or palate are common birth defects of complex etiology. Cleft lip, with or without cleft palate, occurs more commonly than cleft palate alone and is the most common of the signi?cant orofacial anomalies. It can occur in isolation or as part of a broad range of syndromes. They occur with a worldwide frequency of 1 in 700 (24). Commissural lip pits were estimated to be present in 17.4% of the population in Israeli Jews of different ethnic origin (25).

Malignancies were reported in 22 patients with a prevalence rate of 2.4%. Lip cancer is the most frequent malignant neoplasm of the oral cavity mainly in the tropical countries, with approximately 25% of all oral tumors being carcinomas of the lip (26). While the incidence of lip cancers is low [1-2%], over 90% of these tumors consist of squamous cell carcinomas with the lower lip more commonly involved and, in lesser numbers, basal-cell carcinomas, which generally occur in the upper lip (27). Carcinomas of the lower lip are seen most commonly in male smokers working in the open air, such as sailors, fishermen and farmers. The association of HSV2, exposure to UV rays and other chemical factors considerably increase the risk of these malignancies (28). Bouquot *et al.* (7) reported a prevalence of 0.8/1,000 persons for squamous cell carcinoma and 0.04/1,000 persons for basal cell carcinomas.

Pigmented lesions such as hemangiomas were observed in only 4 patients. Bouquot *et al.* (7) observed a prevalence of 3.7/1,000 persons for hemangiomas. Hemangiomas are benign vascular tumors commonly occurring in infancy and childhood, with few present from birth or even developing in adults. 60% of the lesions occur in head and neck region with lip, tongue, and palate being the most preferred site (29). Other lesions observed in this study were in association with immunological disorders [0.2%] such as systemic lupus erythematosus; lesions that resulted from allergic conditions were observed in 1.4% of the population and benign tumours such as fibroma and papilloma were observed in 10 patients.

The findings of this study show that the prevalence of lip lesions in an Indian population is 18.8%. Due to the high prevalence of these lesions in the general population, dental clinicians should be aware of the clinical appearance, etiology, diagnosis and the required treatment for the lesion. A number of risk factors have been associated with these lesions, such as poor oral hygiene, tobacco and alcohol consumption, age, and underlying systemic conditions which may have certain oral manifestations.

## Conclusions

The present study is the first study to report the prevalence of various lesions of the lip in India. But due to the lack of similar studies in the Indian subcontinent, no conclusion can be drawn regarding the prevalence in this region. India has a vast geographic area, which differ with regard to the socioeconomic, educational, cultural and behavioral traditions. These factors may affect the oral health status. Hence, a nationwide study is required to obtain nationwide representative data. Early and correct diagnosis and treatment is challenging for most clinicians because of the wide variety of disease processes that can present with similar appearing lesions and the fact that most clinicians receive inadequate training in labial diseases.
